# TGFβ2 Induces the Soluble Isoform of CTLA-4 – Implications for CTLA-4 Based Checkpoint Inhibitor Antibodies in Malignant Melanoma

**DOI:** 10.3389/fimmu.2021.763877

**Published:** 2022-01-05

**Authors:** Rahul C. Khanolkar, Chu Zhang, Farah Al-Fatyan, Linda Lawson, Ivan Depasquale, Fiona M. Meredith, Frank Muller, Marianne Nicolson, Lekh Nath Dahal, Rasha Abu-Eid, Sanjay Rajpara, Robert Norman Barker, Anthony D. Ormerod, Frank James Ward

**Affiliations:** ^1^Institute of Medical Sciences, School of Medicine, Medical Sciences and Nutrition, University of Aberdeen, Aberdeen, United Kingdom; ^2^Burnside House, Aberdeen Royal Infirmary, Aberdeen, United Kingdom; ^3^Ward 214 Plastic Reconstructive Surgery & Burns Unit, Aberdeen Royal Infirmary, Aberdeen, United Kingdom; ^4^Anchor Unit – Clinic D, Aberdeen Royal Infirmary, Aberdeen, United Kingdom; ^5^Institute of Translational Medicine, Medical Research Council (MRC) Centre for Drug Safety Science, University of Liverpool, Liverpool, United Kingdom; ^6^Institute of Dentistry, School of Medicine, Medical Sciences and Nutrition, University of Aberdeen, Aberdeen, United Kingdom

**Keywords:** checkpoint inhibitor, CTLA-4 (cytotoxic T lymphocyte-associated antigen 4), sCTLA-4, melanoma, TGFβ2, T cells

## Abstract

Malignant melanoma is an aggressive form of cancer, which can be treated with anti-CTLA-4 and anti-PD-1 checkpoint inhibitor antibodies but while anti-CTLA-4 antibodies have clear benefits for some patients with melanoma, productive responses are difficult to predict and often associated with serious immune related adverse events. Antibodies specific to CTLA-4 bind two major isoforms of CTLA-4 in humans, the receptor isoform and a second naturally secretable, soluble isoform - sCTLA-4. The primary aim here was to examine the effect of selectively blocking the function of sCTLA-4 on *in vitro* immune responses from volunteer healthy or melanoma patient PBMC samples. Addition of recombinant sCTLA-4 to healthy PBMC samples demonstrated sCTLA-4 to have immunosuppressive capacity comparable to recombinant CTLA4-Ig, partially reversible upon antibody blockade. Further, we identified a mechanistic relationship where melanoma patient TGFβ2 serum levels correlated with sCTLA-4 levels and provided the basis for a novel protocol to enhance sCTLA-4 production and secretion by T cells with TGFβ2. Finally, a comparison of selective antibody blockade of sCTLA-4 demonstrated that both healthy and melanoma patient effector cytokine responses can be significantly increased. Overall, the data support the notion that sCTLA-4 is a contributory factor in cancer immune evasion.

## Introduction

Malignant melanoma is a dangerous form of skin cancer, whose incidence has risen over the last thirty years. In 2020, global incidence of melanoma reached 324,635 cases with 57,043 deaths (1). Treatment of primary lesions, which emerge from hyperproliferation of melanocytes, is usually performed by resection and offers a favourable prognostic outcome, whereas metastatic malignant melanoma is very difficult to treat effectively and has a very poor prognosis with a 5-year survival rate of only 22.5% compared with >98% for stage I disease. Recently though, the outcome for such patients has been improved by the introduction of novel checkpoint inhibitor therapies that target immune checkpoint receptors including CTLA-4, PD-1 and PD-L1, to promote anti-tumor immunity (2).

The inhibitory CTLA-4 receptor (3, 4), expressed primarily on regulatory (Treg) and activated effector T cells, was the first target for checkpoint inhibitor immunotherapy (5) and antibodies that target the CTLA-4 receptor allow priming and expansion of tumor-specific T cell responses by promoting CD28 mediated T cell costimulation (6). Ipilimumab, a fully human IgG_1_ anti-CTLA-4 antibody, has been approved as a monotherapy for the treatment of advanced malignant melanoma (7) and together with anti-PD-1 mAb, nivolumab, for the treatment of metastatic melanoma, renal cell carcinoma and metastatic colorectal cancer (8–10).

The introduction of ipilimumab in 2011 (7) provided a tangible improvement in metastatic melanoma patient outcomes, and for some individuals an enduring remission from disease, but there remains scope for improving the therapy further. Currently, there is no biomarker for effective stratification of patient responders, or as a guide for severe immune-related adverse effects associated with the therapy. Retrospective analyses of patients treated with ipilimumab as a monotherapy also indicate that approximately only 22% of patients benefit from long term disease remission (11). In contrast, more recent checkpoint inhibitor antibodies that disrupt interaction between PD-1 on T cells (12, 13) and PD-L1 (14–16) on tumor cells have been approved for a broader range of cancers but also demonstrate improved patient response frequency and safety compared with ipilimumab (17). This latter approach is aided by using PD-L1 tumor expression levels as a stratification biomarker to identify patients most likely to respond to the therapy (18).

While there is no doubt about the potential therapeutic benefits of ipilimumab, its precise mechanism of action remains to be fully resolved, as does the biology of CTLA-4 more generally. CTLA-4 is crucial at the priming stage of naïve anti-tumor T cell responses by professional antigen presenting cells (APC), and therefore its blockade has the potential to generate *de novo* powerful CD8^+^ cytotoxic and CD4^+^ helper T cell anti-tumor effector responses. Despite its fundamental importance to naïve T cell differentiation and activation, the clinical response variation between patients receiving anti-CTLA-4 therapy hints that other processes such as differences in tumour immunogenicity, the manifestation of tumour neoantigens and other immune factors may also be important for the generation of effective responses (19, 20). Anti-CTLA-4 antibodies may also function by directly binding to and depleting Treg *via* macrophage dependent antibody-dependent cell-mediated cytotoxicity (ADCC) within the tumor microenvironment (21, 22). This uncertainty of mechanism is reflected in the unusual kinetics of anti-CTLA-4 mAb therapy, in which for some patients, tumors may grow for weeks or months after therapy commences, before eventually stabilising, shrinking and ultimately disappearing altogether. No one hypothesis adequately explains this extended hiatus between therapeutic intervention and productive anti-tumor immunity.

Despite a primary focus on the CTLA-4 receptor, anti-CTLA-4 antibodies bind two isoforms of CTLA-4 in humans, the full length CTLA-4 receptor and a second secretable form of CTLA-4, soluble CTLA-4 (sCTLA-4) (23, 24). Soluble CTLA-4 is an alternatively spliced variant of full length CTLA-4 in which the transmembrane domain, encoded by exon 3, is not utilised and a frameshift mismatch during post-transcriptional splicing of exon 2 (extracellular domain) to exon 4 (cytoplasmic domain) gives rise to a unique C terminal amino acid sequence that replaces the cytoplasmic domain of full length CTLA-4 (24). Soluble CTLA-4 like its full-length counterpart can bind to B7 (CD80/CD86) ligands on APC (24).

The secretory isoform of CTLA-4 was identified after the receptor isoform and less is known regarding any immunoregulatory properties it may possess. Few studies that aim to explain response diversity in patients receiving anti-CTLA-4 therapy have examined sCTLA-4 as a significant influential factor (25, 26). Analysis of sCTLA-4 serum levels in patients with acute B lymphoblastic leukaemia (B-ALL) (27), malignant melanoma (28) or mesothelioma (29), however, revealed increased production together with good evidence that tumour cells may actually be secreting this potentially immunosuppressive molecule. Retrospective analyses of patients treated with ipilimumab also discovered that patients with higher serum levels of sCTLA-4 were significantly more likely to respond to the therapy than those with low levels (25). Further support for the clinical relevance of sCTLA-4 to cancers in particular, has emerged with the discovery that the repulsive guidance molecule B (RGMB) acts as a ligand for sCTLA-4 and greatly strengthens its immunosuppressive activity through enhanced CD80 binding (30).

The TGF-β family includes three closely related molecules, TGFβ1, 2 and 3 whose roles in maintaining immune homeostasis are crucial, but which also play a complex role in tumour development (31, 32). Despite initial anti-tumor growth effects, during tumorigenesis TGFβ can both assist the metastatic EMT transition process and promote evasion from anti-tumour immunity (33–35). Thus TGFβ represents another potential target for anti-tumour immunotherapy and could be used in combination with anti-CTLA-4/PD-1/PD-L1 antibodies (31).

Previously, we demonstrated that selectively blocking sCTLA-4 boosted antigen-specific immune responses and further, generated effective anti-tumour immunity in the B16F10 model of metastatic melanoma, which was comparable to that achieved with pan-specific anti-CTLA-4 mAb (36). Our initial analysis of sCTLA-4 production identified Tregs to produce and secrete this immunoregulatory molecule. Here, we demonstrate recombinant sCTLA-4 to be directly immunosuppressive with regard to T cell responses and identify a novel functional association between sCTLA-4 production and the immunoregulatory cytokine, TGFβ2. Further, by selectively targeting this particular isoform, melanoma patient derived PBMC response activity *in vitro* was increased significantly with higher production of the T cell effector cytokines IFN-γ and IL-17A when compared with non-selective ipilimumab. We propose a new mechanism through which tumor cells utilise TGFβ2 to promote production of sCTLA-4 as a novel mechanism of immune evasion and further, highlight the urgent need to examine the relevance of sCTLA-4 to immune checkpoint inhibitor therapy.

## Methods

### Donors, Ethics and PBMC Sample Preparation

Blood samples were collected by venepuncture from patients at the dermatology, oncology or plastic surgery clinics at Aberdeen Royal Infirmary (see **Table 1** for demographic summary) and compared with age and sex-matched healthy volunteer donors (n=27). Sera from the Lupus patient cohort was provided from a previous study, which was age but not sex matched (n=40) (37). PBMC were prepared using Lymphoprep 1.077 (Axis Shield, Dundee, UK) density gradient centrifugation and cultured essentially in RPMI 1640 medium (Thermofisher Scientific, Paisley, UK) supplemented with 5% autologous human serum in an atmosphere of 37°C, 5% CO_2_ as previously described (38). 1×10^6^ PBMC were cultured for 5 days in 1 mL wells unless otherwise stated. Jurkat T cells (1×10^6^ per well) were stimulated for 48 hours with PHA-L according to manufacturer’s instructions and B7.1/2Ig at 1 μg/mL (Peprotech EC, London, UK) in an atmosphere of 37°C, 5% CO_2_. Spleens from Balb/c mice were obtained from the University of Aberdeen, Medical research facility and stimulated in the presence of anti-CD3 antibody (Clone: 2C11, BD Biosciences, Oxford, UK), murine TGFβ2 (R&D Systems, Abingdon, UK) and IL-2 (Peprotech EC) for 8 days.

**Table 1:** Summary of the melanoma patient demographics.

Demographic and clinical characteristics	Melanoma patients
Age, years	57.3 ± 36.1
Sex, no. female/male	18/12
Average disease duration (months)	36 ± 2
Recruiting clinic	
- Oncology	9
- Dermatology	22
- Plastic surgery	1
Average Breslow thickness (range mm)	2.0 (0 – 10.0)

### Cytokine and sCTLA-4 ELISA

ELISA for cytokines in cell culture supernatants or sera was based on previously published methods. Antibody pairs used for the human IL-10 ELISA were clones JES3-19F1 and JES3-12G8 from BD Biosciences), for anti-IFN-γ (clones NIB42 and 4S.B3 BD Biosciences, Oxford, UK), and for anti-IL-17A (clones eBio64CAP17 and eBio64DEC17, Thermofisher Scientific). Cytokine standards were from Peprotech EC Ltd. Bound antibody was detected using streptavidin-labelled alkaline phosphatase with a phosphate substrate (both Sigma Aldrich, Gillingham, UK), and absorbance measured at 450nm (corrected with a reference reading at 492nm) with a Multiskan MS microplate photometer (Life and Laboratory Sciences, Basingstoke, UK). IL-2 was measured using an IL-2 Human Uncoated ELISA Kit with Plates (Thermofisher) according to manufacturer’s instructions.

The selective ELISA for human sCTLA-4 used the anti-CTLA-4 murine mAb clone BNI3 (2 µg/ml) as a capture reagent and biotinylated mAb clone 73-B1 (IgG1κ) as the sCTLA-4 specific detection reagent using the same protocol described for the cytokine ELISA above. The alternatively spliced recombinant human sCTLA-4 (MRC PPU Services, University of Dundee, Dundee) was used to construct standard curves. Biotinylated anti-sCTLA-4 clone 73-B1 cross-reacts with murine sCTLA-4 and was used in ELISA to detect the presence of murine sCTLA-4 together with a capture anti-murine CTLA-4 mAb (clone: 4F10, BD Biosciences).

### Cancer Cell Lines

Adherent malignant melanoma epithelial cell lines G-361 (CRL-1424) and A-375 (CRL-1619) were obtained from the American Tissue culture collection (LGC standards, London, UK) and cultured in McCoy’s 5a Medium Modified Medium supplemented with 10% Foetal bovine serum (FBS; G-361) or Dulbecco’s Modified Eagle’s Medium +10% FBS (A-375) according to protocols provided by ATCC.

### Confocal Microscopy

For intracellular analysis of CTLA-4 and sCTLA-4 by confocal microscopy, G-361 and A-375 cell lines were prepared according to ATCC protocols and seeded into 24 well plates containing sterile coverslips coated with poly-l-lysine to aid adherence. Cells were allowed to grow to 80-90% confluence before fixing with 4% paraformaldehyde (BD Cytofix) for 10 min at RT, treatment with 0.3M glycine in PBS to prevent background fluorescence (10 min at RT), permeabilization with 0.2% Triton X-100 in PBS (5 min at RT) and blocking with 1% BSA in PBS. Cells were washed three times (2 minutes per wash) between each step. The cell lines were then incubated with biotinylated anti-sCTLA-4 mAb, JMW-3B3 or a non-specific biotinylated IgG1 antibody (Thermofisher; both 20 μg/mL) and incubated O/N at 4°C. The cell lines were subsequently washed and incubated with rabbit polyclonal anti-CTLA-4 antibody before staining with Streptavidin-AF555 to selectively reveal sCTLA-4 and Goat anti-Rabbit IgG AF488 to reveal total CTLA-4 (CTLA-4 receptor and sCTLA-4), each according to manufacturer’s instructions. Finally, coverslips were placed on glass slides and mounted with Prolong Gold anti-fade mountant containing DAPI (Fisher Scientific) before analysis with a Leica LSM710 confocal microscope at the Institute of Medical Sciences, Microscopy and Histology Core Facility. Images were analysed using ‘ZEN BLUE’ software.

### Flow Cytometry Analysis

For surface staining of markers using flow cytometry, following the appropriate stimulation protocol, cells were first washed twice with staining media (PBS + 0.5% BSA) and stained with fixable viability dye eFluor 780 as per the manufacturer’s guidance. The cells were washed with staining media and suspended in blocking buffer (PBS + 5% BSA) at 4°C for 30 minutes. The cells were stained with the appropriate fluorochrome conjugated antibodies at 4°C for 30 minutes and fixed with BD Cytofix fixation solution (BD Biosciences) for 10 minutes at RT.

To measure phosphorylation of intracellular molecules, following T cell stimulation with plate bound anti-CD3 mAb (1 μg/mL), the cells were washed and treated with BD Cytofix/Cytoperm fixation/permeabilization solution (BD Biosciences) to arrest intracellular phosphorylation and subsequent activation of cells. The optimum time point for measurement was identified as 10 minutes following stimulation following a “sighting” experiment to determine the optimum time for assessment (**Figure 2A**). The cells were washed, blocked and stained for surface and intracellular molecules according to the protocol outlined above together with appropriate isotype controls. All fluorochrome conjugated antibodies specific to surface and intracellular molecules were acquired from BD biosciences. For all flow cytometry readouts, a minimum of 100,000 events were acquired using a BD LSR II flow cytometer (BD Biosciences) and analysed using FlowJo analysis software.

### Statistics

Power calculations: For the main patient study and based on healthy donor PBMC sample responses to stimulation in the presence or absence of sCTLA-4 antibody blockade in which analysis of 20 samples provided statistically significant deviation from the null hypothesis, a comparative analysis of responses from 20 melanoma patient blood samples provided approximately 80% power to detect a 65% difference in responses at the 5% significance level. Differences between treatments were analyzed with an Anova one-way test with Tukey *post-hoc* analysis, Mann-Whitney U, two-tailed, unpaired t test or Pearson’s r tests as outlined in figure legends. Where used, Bar and whisker plot error bars show the minimum and maximum value for each cohort.

### Study Approval

Written informed consent was obtained from all donors. Ethical approvals were obtained from East of Scotland Research Ethics Committee (ref:13/NS/0126 & 10/S1401/20) and the study was performed and archived according to the protocols provided by the East of Scotland Research Ethics Committee. Exclusion criteria: Potential volunteer donors were included in the study if over the age of 18, independent of melanoma disease status from the dermatology, plastic surgery and oncology clinics at the University of Aberdeen. All potential volunteer candidates were provided with a patient information sheet prior to providing a blood donation. Patient weight was not recorded.

## Results

### Immunosuppressive Properties of sCTLA-4

Most studies involving sCTLA-4 have used pan-specific anti-CTLA-4 antibodies, but here, we have used two selective anti-sCTLA-4 monoclonal antibodies, JMW-3B3 (IgG1λ) (36) and 73-B1 (IgG1κ), together with recombinant (rec.) sCTLA-4 to examine the immunoregulatory properties of sCTLA-4 primarily in melanoma patient donor samples. Both antibodies were raised against the unique C terminal sequence of sCTLA-4 and neither cross-react with the CTLA-4 receptor.

**Figure 1:**
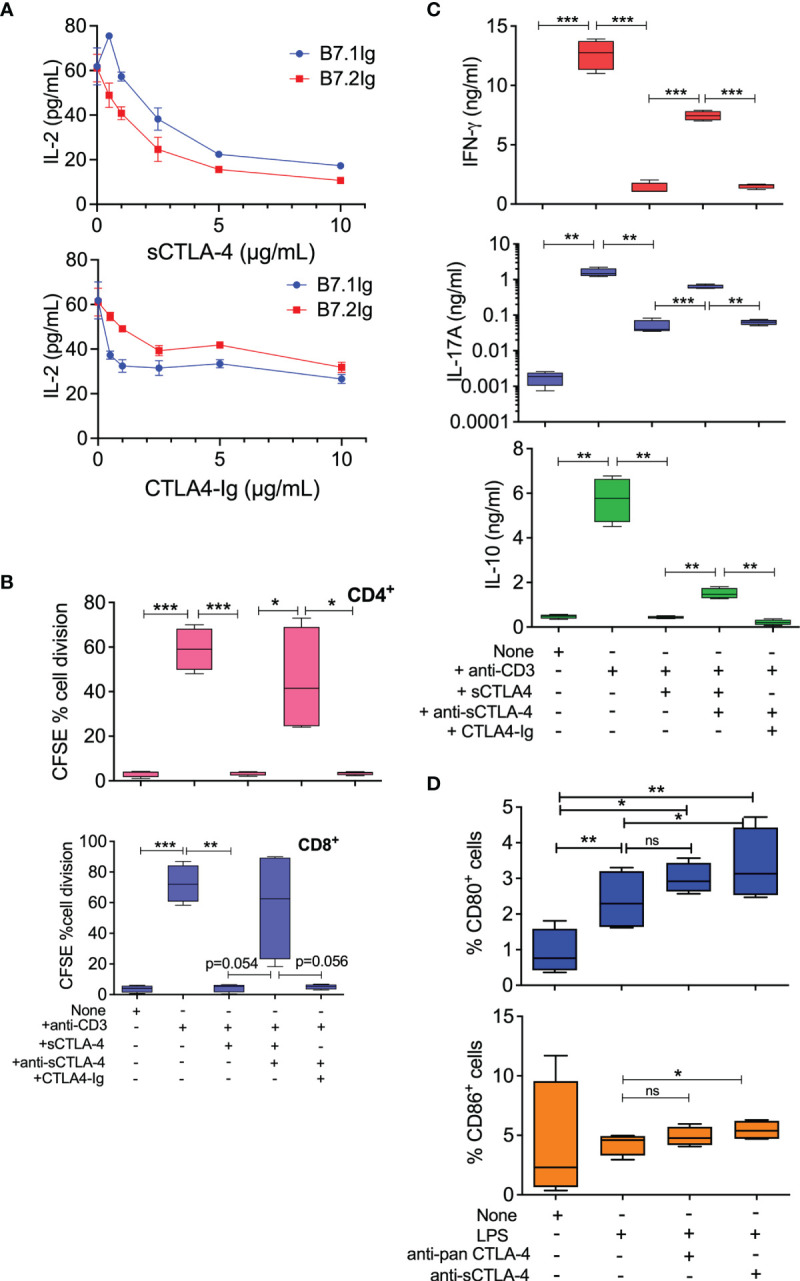
Immunoregulatory properties of recombinant human sCTLA-4. **(A)** Comparison of immunosuppressive activity of rec. sCTLA-4 with CTLA4-Ig in Jurkat T cell cultures stimulated with PHA-L and either B7.1Ig or B7.2Ig costimulatory ligands for 48 hours at 37°C, 5% CO2 (Mean (SD); representative of n = 4). **(B, C)** Donor PBMC were stimulated with anti-CD3 mAb for 5 days at 37°C, 5% CO2 in the presence of human rec. sCTLA-4, CTLA4-Ig ± anti-sCTLA-4 antibody, 73-B1, before analysis of cell division by flow cytometry **(B)** and of cell culture supernatant cytokine levels by ELISA (**C**, n=5). **(D)** Effect of anti-CTLA-4 or sCTLA-4 antibody blockade on PBMC derived CD11c^+^ APC CD80/CD86 T cell levels by flow cytometry. Cells were stimulated with 1 mg/mL LPS for 18 hrs in the presence or anti-sCTLA-4 (73-B1) or pan anti-CTLA-4 mAb (BNI3; both 10 μg/mL; n=4). (One-way Anova with Tukey *post-hoc* multiple comparison analysis, *P < 0.05 **P < 0.01, ***P < 0.001, ns, not significant).

Previous reports have indicated sCTLA-4 to be immunosuppressive because selective removal from cell cultures can boost antigen-specific effector T cell responses (36). To determine whether or not sCTLA-4 is directly immunosuppressive, we added rec. human sCTLA-4 (sCTLA-4) or CTLA4-Ig to Jurkat T cells stimulated with PHA-L and either B7.1Ig or B7.2Ig costimulatory ligands before measuring IL-2 cell culture supernatant levels by ELISA after 48 hours (**Figure 1A**). Both reagents were comparatively immunosuppressive and demonstrated the suppressive capacity of natural sCTLA-4. We also assessed the immunosuppressive effects of sCTLA-4 in normal healthy donor PBMC cell cultures stimulated with plate-bound anti-CD3 mAb for 5 days and examined its effects on cell division by flow cytometry and T cell cytokine production by ELISA (**Figures 1B, C**). Addition of rec. human sCTLA-4 at 10 μg/ml was immunosuppressive, significantly decreasing both CD8^+^ and CD4^+^ T cell proliferation and PBMC culture supernatant levels of IFN-γ, and IL-17A and IL-10. The immunosuppressive effects of sCTLA-4 were significantly reversed by the addition of anti-sCTLA-4 mAb, 73-B1, demonstrating a sCTLA-4 specific effect. CTLA4-Ig also suppressed these PBMC responses, but its immunosuppressive effects were not reversible by anti-sCTLA-4 mAb, 73-B1, as CTLA4-Ig lacks the C terminal epitope recognised by the antibody, which is present on natural sCTLA-4.

Analysis of human healthy donor CD11c^+^ antigen presenting cells stimulated with LPS, within the PBMC population, revealed that selective anti-sCTLA-4 antibody blockade also significantly increased detectable cell surface levels of CD80/CD86 (**Figure 1D**), while anti-panCTLA-4 antibody was slightly less effective. Thus, sCTLA-4 has direct immunosuppressive effects on activated PBMC by blocking or inhibiting costimulatory interactions and its removal enhances effector T cell activity.

### Selective Antibody Blockade of sCTLA-4 Does Not Suppress Phosphorylation of T Cell Signalling Proteins Slp76 and ZAP-70

**Figure 2:**
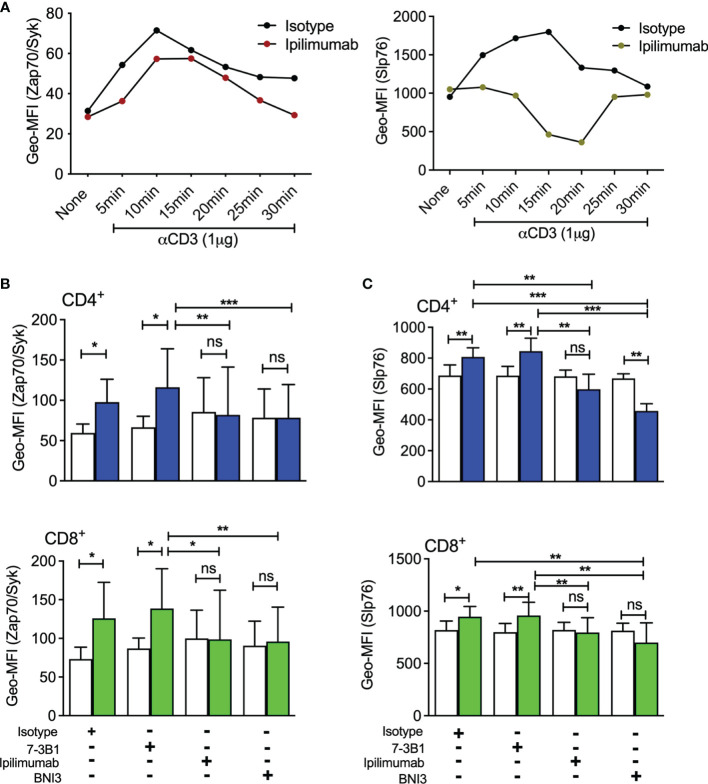
Analysis of sCTLA-4 blockade on T cell receptor phosphorylation levels **(A)** Sighting experiment to evaluate the optimum period for measuring phosphorylation levels of Slp76 and ZAP-70 following stimulation of T cells with anti-CD3 Mab (n = 2). Effect of anti-CTLA-4 or anti-sCTLA-4 mAb blockade on phosphorylation levels of T cell signalling components **(B)** ZAP-70 and **(C)** Slp76 following activation. Healthy donor PBMC were stimulated with anti-CD3 mAb for 15 minutes, fixed and CD4^+^ and CD8^+^ T cells were analysed for ZAP-70 and Slp76 levels of phosphorylation by flow cytometry. White bars represent an IgG1 (Zap-70) or an IgG2a (Slp76) isotype control, while green/blue bars represent specific antibody. n=5; *P < 0.05 **P < 0.01, ***P < 0.001, ns, not significant, P values determined by Student t test; Mean (SD) values are shown).

Previously, we demonstrated that selective blockade of sCTLA-4 significantly enhanced antigen-specific CD4 T helper T cell responses compared with pan anti-CTLA-4 antibody blockade (36). To further examine the effects of anti-CTLA-4 mAbs on T cell activation, we stimulated PBMC with anti-CD3 mAb and assessed the effects by phosphoflow cytometry of anti-sCTLA-4 vs. pan anti-CTLA-4 antibody blockade on phosphorylation levels of both T cell receptor signalling cascade molecules Slp76 and ZAP-70 and compared to an isotype control antibody (**Figure 2**, white bars isotype, green/blue bars specific antibody). Stimulation of donor-derived T cells isolated from PBMC with anti-CD3 mAb, as expected, induced an increase in phosphorylated Slp76 and ZAP-70 in both CD4^+^ and CD8^+^ T cells compared with resting cells, and addition of anti-sCTLA-4 mAb clone 73-B1 did not affect phosphorylation levels. This is not surprising because anti-sCTLA-4 mAbs do not interact directly with cells. Addition of anti-CTLA-4 mAbs BNI3 or ipilimumab, however, completely abolished any increase in phosphorylation in both anti-CD3 mAb stimulated CD4^+^ and CD8^+^ T cells compared with resting cells.

In summary, we find that selective targeting of sCTLA-4 has no inhibitory effect on phosphorylation levels of ZAP-70 and Slp76 after activation of CD4^+^ and CD8^+^ T cells.

### A Mechanistic Relationship Between sCTLA-4 and TGF β2

**Figure 3:**
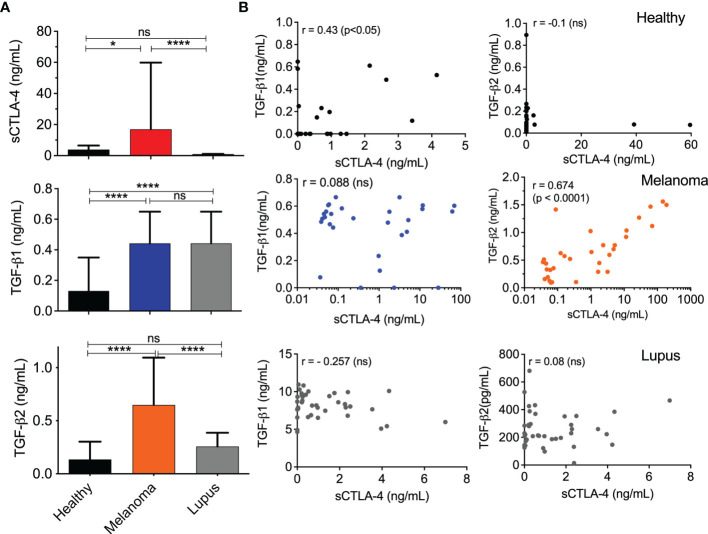
Correlation analysis of TGFβ and sCTLA-4 serum levels in melanoma, lupus, and healthy volunteer donor cohorts. **(A)** Serum levels of TGFβ1, TGFβ2 and sCTLA-4 in healthy (n=27), melanoma patient (n = 32) and lupus patient donor cohorts (n = 40). (Mean (SD) *P < 0.05, ****P < 0.0001, ns, not significant, P values determined by one-way ANOVA test). **(B)** Regression analysis of TGFβ1 or TGFβ2 with sCTLA-4 levels in sera from healthy, melanoma patient and lupus patient donor cohorts (Pearson’s r test, p values shown in figure).

Previous reports indicated that serum levels of sCTLA-4 are raised in some cancers including malignant melanoma, but those studies did not use sCTLA-4 selective antibodies and were, therefore, unable to distinguish fragments of CTLA-4 receptor cleaved from cell surfaces from native alternatively spliced sCTLA-4. We analysed and compared melanoma patient serum sCTLA-4 levels with those from healthy or lupus donor volunteers (**Figure 3**). Serum levels of sCTLA-4 were significantly higher in the melanoma patient cohort compared with both healthy and lupus patient volunteer donor serum cohorts.

The immunosuppressive TGFβ cytokine isoforms contribute to immune evasion strategies promoted by tumors and in a number of cancers, TGFβ2 has been closely implicated in epithelial to mesenchymal transition (EMT), a precursor to the metastatic process (39). We examined and compared serum levels of both TGFβ1 and 2 in the same healthy, melanoma and lupus cohorts (**Figure 3**). The lupus patient serum cohort was examined to determine whether any correlation between sCTLA-4 and TGFβ serum levels would be replicated in the context of an autoimmune disease. Serum levels of TGFβ1 were significantly raised in both the melanoma and lupus patient serum cohorts compared with healthy donors (**Figure 3A**), but TGFβ2 serum levels were significantly higher solely in the melanoma patient cohort. Correlative analysis of TGFβ1, TGFβ2 and sCTLA-4 levels in the three donor serum cohorts (**Figure 3B**) revealed a significant positive correlation between TGFβ2 and sCTLA-4 serum levels restricted solely to the melanoma serum cohort, and a weaker but significant correlation between TGFβ1 and sCTLA-4 in the healthy donor serum cohort. No other correlation was observed.

**Figure 4:**
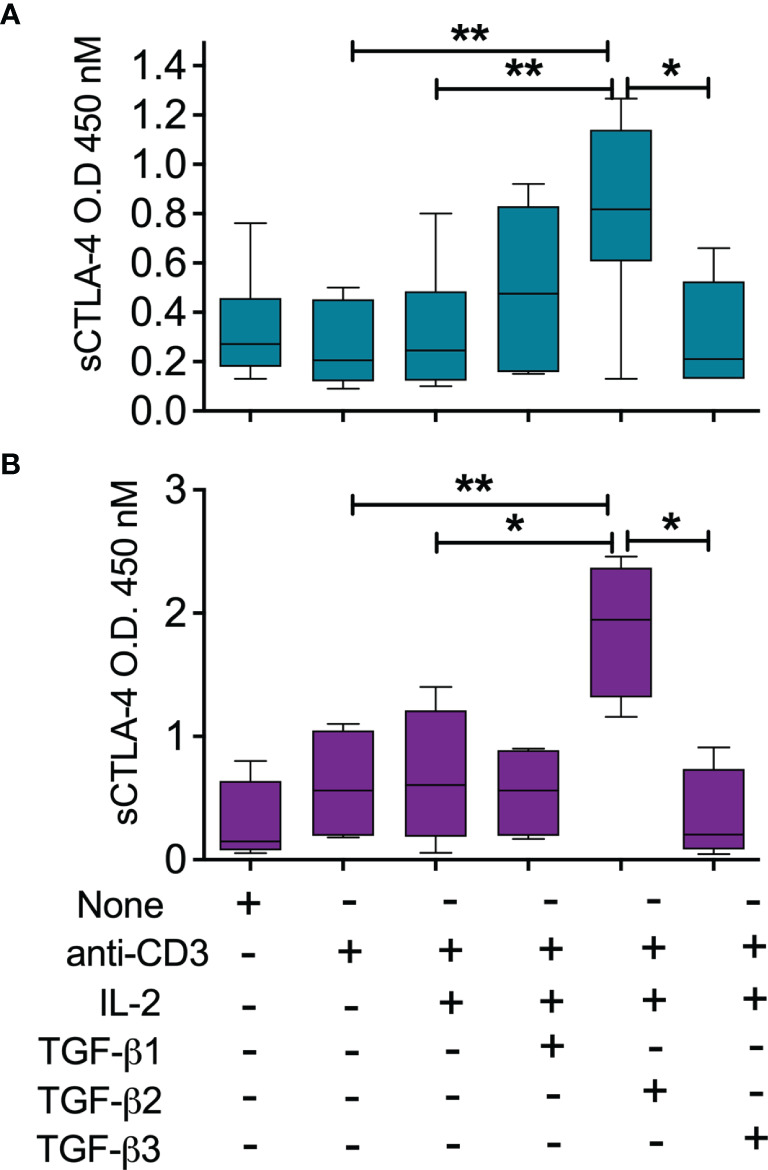
Induction of sCTLA-4 by TGFβ2. **(A)** PBMC were isolated from healthy donors (n = 5, top panel) or **(B)** Balb/c mice (n = 4, lower panels) were incubated for 8 days after stimulation with anti-CD3 mAb (0.1 μg/mL), IL-2 (20 ng/mL) in the presence of TGFβ isoforms 1,2 and 3 each at 20 ng/mL before analysis of cell culture supernatant sCTLA-4. n=5; *P < 0.05 **P < 0.01, P values determined from a one-way test with Tukey *post-hoc* analysis; median values are shown).

The analysis of the melanoma patient serum cohort revealed for the first time a potential relationship between TGFβ2 and sCTLA-4 in melanoma patients and also raised the prospect that TGFβ2 has a role in stimulating the production of sCTLA-4. TGFβ is important for the induction of peripheral Treg (pTreg) and has also been used in protocols to generate inducible Treg (iTreg) *in vitro* for clinical use (40). We fractionated CD3^+^ T cells from healthy donor PBMC and stimulated them with anti-CD3 mAb in the presence of IL-2, while comparing the ability of all three TGFβ isoforms, TGFβ1, TGFβ2 and TGFβ3 to induce sCTLA-4 production (**Figure 4A**). Following an extended incubation of 8 days, sCTLA-4 supernatant levels were significantly increased after incubation with TGFβ2, with levels significantly higher than any other treatment. In some individual donors TGFβ1 was also able to increase sCTLA-4 levels, but collectively this population did not achieve significance. Finally, we repeated this sCTLA-4 induction experiment using BALB/c mouse splenocytes with very similar results (**Figure 4B**). Together, the data demonstrate that sCTLA-4 production can be consistently induced in activated T cells by the immunoregulatory cytokine TGFβ2, supporting the notion that TGFβ2, which is primarily produced by tumor cells in malignant melanoma, has the potential to induce immunosuppressive sCTLA-4 within the tumor environment.

### Melanoma Derived Cell Lines Produce sCTLA-4

**Figure 5:**
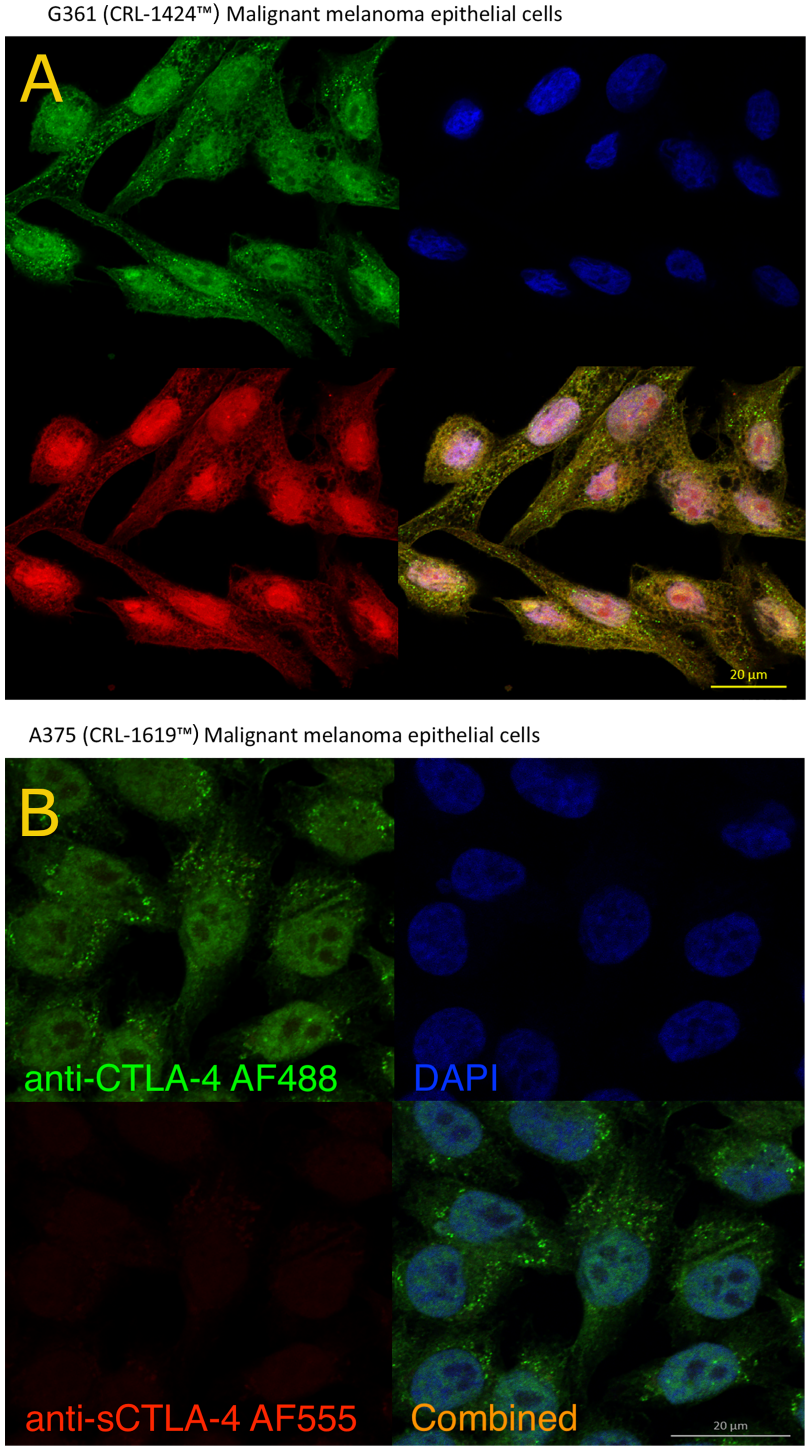
Analysis of CTLA-4 receptor and sCTLA-4 in melanoma cell lines by confocal microscopy. G-361 **(A)** and A-375 **(B)** human melanoma cell lines were incubated on sterile poly-L-lysine coated coverslips until confluence was reached and stained with specific anti-sCTLA-4 mAb (JMW-3B3, AF555 - red) and rabbit polyclonal anti-panCTLA-4 (AF488 – green) together with DAPI (blue). Both antibodies bind sCTLA-4 yielding a yellow/orange color. Representative of n>3 experiments.

Contrary to current perceptions, studies of CTLA-4 in tumor cell biopsies and cell lines have revealed that CTLA-4 and sCTLA-4 may also be actively produced by cancer cells (28–30) perhaps as a means of tumor cell mediated immune evasion. The availability of anti-sCTLA-4 antibodies allowed us to selectively stain and differentiate both full-length CTLA-4 receptor and sCTLA-4 in human melanoma cancer cell lines, as well as those associated with other tumour types. Co-staining with pan anti-CTLA-4 (AF488, **Figure 5**) and anti-sCTLA-4 (AF555) revealed and differentiated the presence of both CTLA-4 receptor and sCTLA-4 in melanoma cell lines. Staining of the G-361 epithelial malignant melanoma cell line revealed sCTLA-4 detectable throughout individual cells, punctuated by CTLA-4 receptor within individual vesicles, by confocal microscopy (**Figure 5A**). Further, while all cancer cell lines tested to date stained positive for the CTLA-4 receptor, not all expressed sCTLA-4, including the A-375 epithelial malignant melanoma cell line (**Figure 5B**; see also **Supplementary Figure 1**).

### Selective Blockade of sCTLA-4 Enhances Effector Cytokine Production by Melanoma Patient PBMC

**Figure 6:**
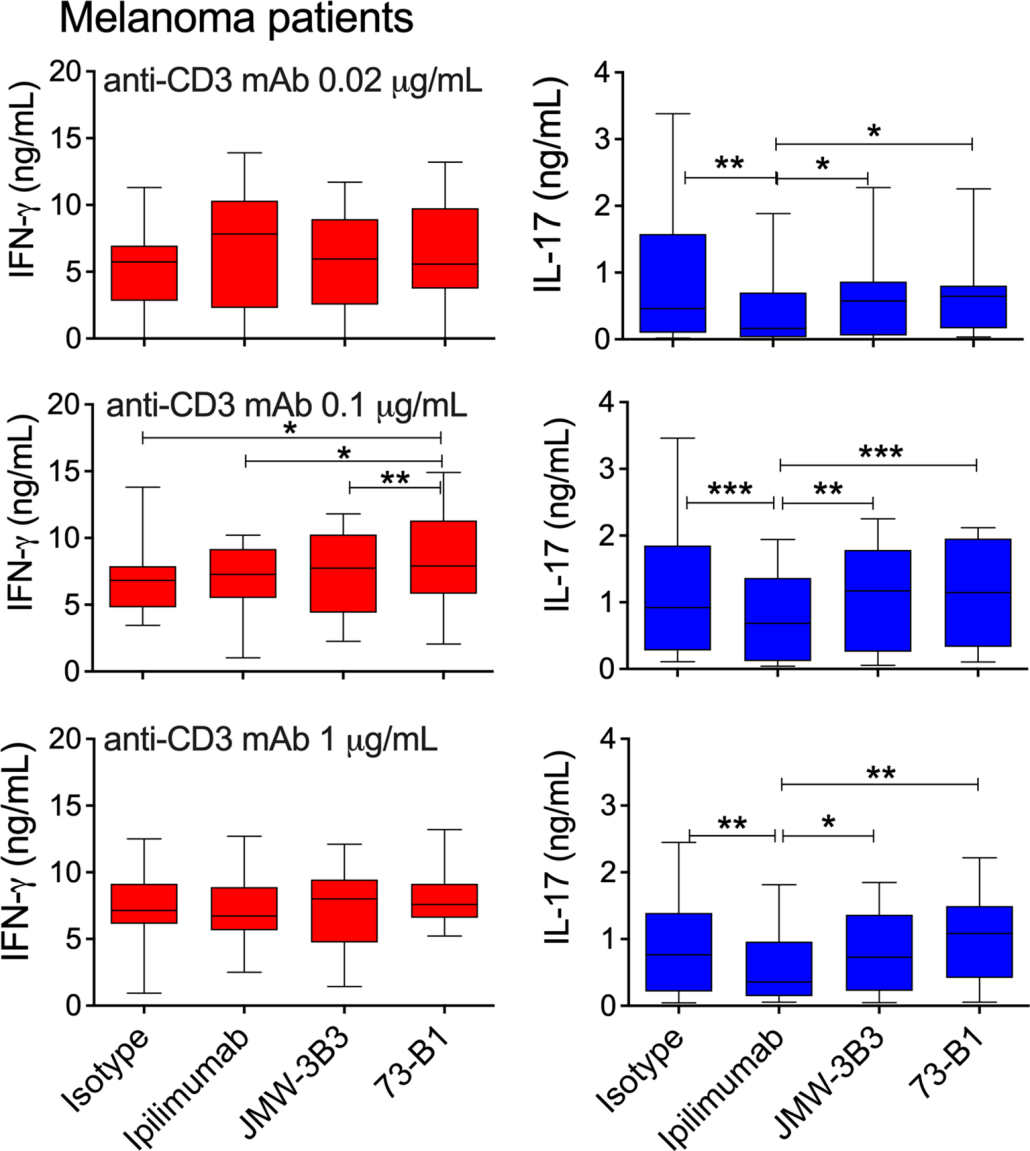
Comparative effect of anti-sCTLA-4 or ipilimumab anti-CTLA-4 mAb blockade on melanoma patient donor PBMC responses to stimulation with 0, 0.1 or 1 μg/mL anti-CD3 mAb. PBMC were stimulated for five days at 37°C 5%CO2 in the presence of plate-bound anti-CD3 and 10 μg/mL IgG1 isotype control, anti-sCTLA-4 mAbs JMW-3B3 and 73-B1, and anti-CTLA-4 mAb, ipilimumab. Cell culture supernatants were measured by ELISA for levels of IFN-γ and IL-17A. n=12; *P < 0.05 **P < 0.01, ***P < 0.001, P values determined from a one-way test with Tukey post-hoc analysis).

**Figure 7:**
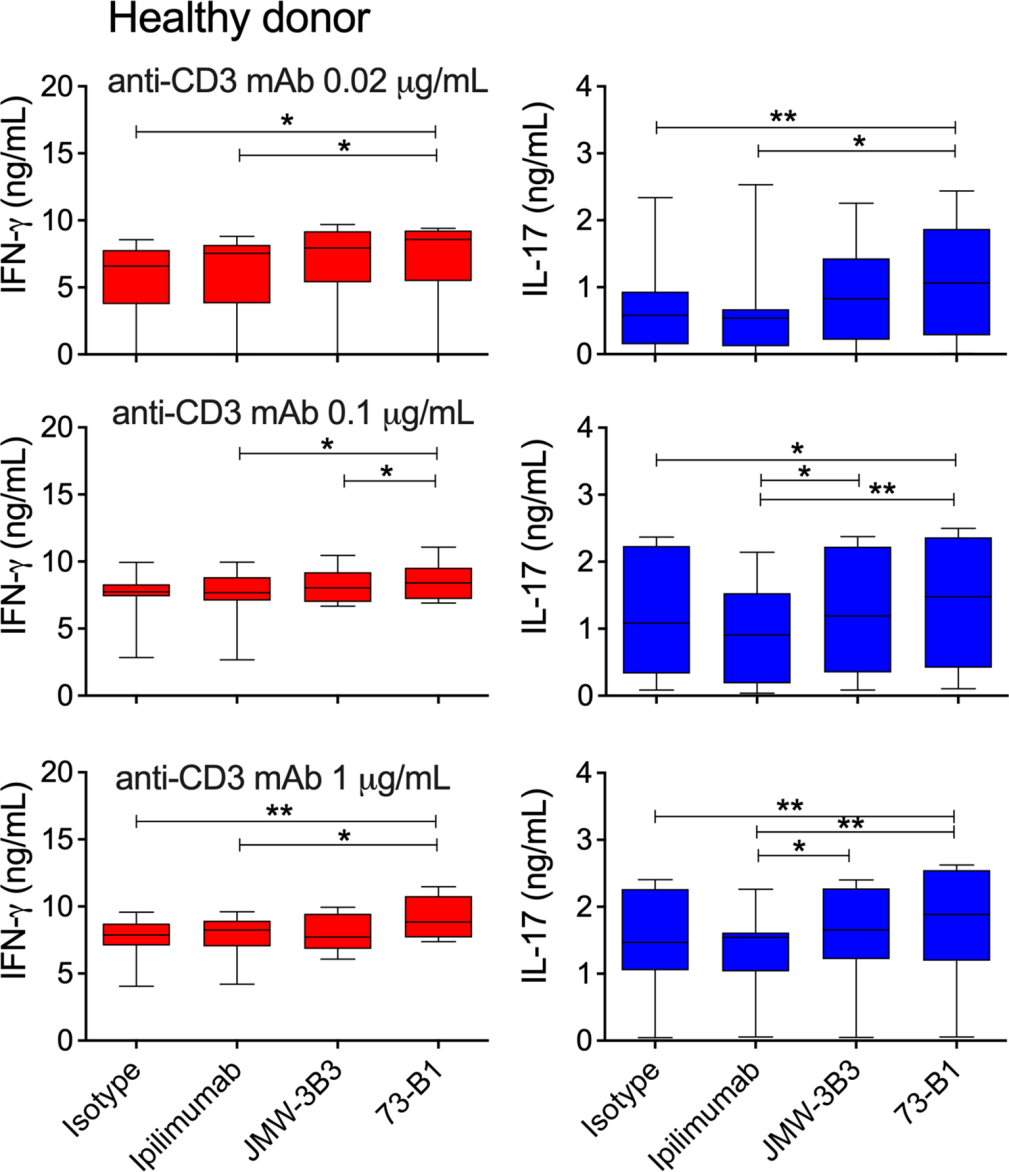
Comparative effect of anti-sCTLA-4 or ipilimumab anti-CTLA-4 mAb blockade on healthy donor PBMC responses to stimulation with 0, 0.1 or 1 μg/mL anti-CD3 mAb. PBMC were stimulated for five days at 37°C 5%CO_2_ in the presence of plate-bound anti-CD3 and 10 μg/mL IgG1 isotype control, anti-sCTLA-4 mAbs JMW-3B3 and 73-B1, and anti-CTLA-4 mAb, ipilimumab. Cell culture supernatants were measured by ELISA for levels of IFN-γ and IL-17A. n=8; *P < 0.05 **P < 0.01, P values determined a one-way test with Tukey *post-hoc* analysis).

In a previous study, we demonstrated that selective blockade of sCTLA-4 was as effective as pan-CTLA-4 blockade at reducing lung tumor frequency in the B16F10 model of melanoma lung metastasis (36). Thus, we examined the selective effects of anti-sCTLA-4 antibody blockade compared directly with anti-CTLA-4 mAb ipilimumab on supernatant effector cytokine levels from PBMC cell cultures provided by melanoma patient donors and stimulated with doses of plate-bound anti-CD3 mAb ranging from 0.02 to 1 μg/mL (**Table 1** and **Figure 6**). We also compared selective sCTLA-4 vs. pan-CTLA-4 blockade in cell cultures from healthy volunteer donors (**Figure 7**).

Analysis of IFN-γ, IL-17 and IL-10 supernatant levels from 5-day melanoma patient derived PBMC cell cultures (**Figure 6** and **Supplementary Figure 2**), revealed relatively modest differences in IFN-γ supernatant increases between treatments, with significant increases in levels observed only with both anti-sCTLA-4 mAbs JMW-3B3 and 73-B1 anti-sCTLA-4 clones stimulated significant increases in IFN-γ compared with ipilimumab at low levels of anti-CD3 mAb (0.02 μg/mL) stimulation. No other significant differences were detected.

Differences in supernatant levels of IL-17 were more apparent, with selective blockade of sCTLA-4 by antibody clone 73-B1 significantly increasing levels of IL-17 at every anti-CD3 mAb dose. An interesting observation here, was that significant differences between 73-B1 and ipilimumab arose because of increases in IL-17 in 73-B1 treated cultures but also decreases in ipilimumab treated cultures. No differences in IL-10 levels were detected in any of these treatments (**Supplementary Figure 2**).

Analysis of healthy donor PBMC responses also identified anti-sCTLA-4 clone 73-B1 to generate significant increases in IFN-γ and IL-17 supernatant levels in anti-CD3 mAb stimulated cultures compared with ipilimumab or isotype antibody (**Figure 7**). At higher anti-CD3 mAb doses, anti-sCTLA-4 clone JMW-3B3 also enhanced IL-17 but not IFN-γ levels compared with ipilimumab (**Figure 7**). Once again there were no effects on IL-10 levels by either pan-CTLA-4 or sCTLA-4 selective antibody blockade (**Supplementary Figure 2**). The increases in cytokine production from the healthy donor volunteer cohort were very similar to those identified previously (37).

## Discussion

Having previously identified the soluble isoform of CTLA-4 as a candidate regulatory molecule capable of suppressing antigen specific effector T cell responses (36), we demonstrate here for the first time a novel mechanism based specifically on the TGFβ2 isoform that drives sCTLA-4 to be produced at high levels in human patients with melanoma and which we propose is used as a tumor immune evasion strategy. We provide further evidence that sCTLA-4 contributes to immune regulation through T cell suppression with efficacy similar to that of the artificial soluble form of CTLA-4, CTLA4-Ig and selective blockade of sCTLA-4 also enhanced culture supernatant levels of both IFN-γ and IL-17A effector cytokines in anti-CD3 activated PBMC from volunteer melanoma patients.

Although it is clear that CTLA-4 blockade can induce anti-tumor immunity with increased tumor infiltration of cytotoxic effector T cells, most studies do not account for any potential immunoregulatory contribution from the soluble isoform of CTLA-4. Is this second secretory isoform really of no consequence at all to immune regulation or indeed immunotherapy? Generally, sCTLA-4 is produced by resting immune cells including CD8^+^ T cells, regulatory T cells, monocytes and B cells, but is also secreted by some non-immune cells, notably pituitary gland cells (41) and cancer cells (42). Recent work identified sCTLA-4 to be differentially expressed by thirty different cancer types building upon seminal observations of sCTLA-4 expression in melanoma and mesothelioma (28–30). Previous studies have indicated that sCTLA-4 is immunosuppressive, so to examine this more carefully, we produced recombinant human sCTLA-4 and incubated it with healthy donor PBMC stimulated with anti-CD3 mAb or Jurkat T cells stimulated with PHA-L and B7.1-Ig or B7.2-Ig. We demonstrated that sCTLA-4 was immunosuppressive and capable of inhibiting CD4^+^ and CD8^+^ T cell proliferation and cytokine secretion *in vitro*, partially reversible by selective antibody blockade. Further, sCTLA-4 blockade also increased cell surface levels of CD80/86 on CD11c^+^ APC stimulated with LPS although it remains uncertain whether that arises from an increase in expression levels or is simply an effect of removing sCTLA-4 to allow free display of more CD80/CD86 receptors. In these studies, the immunosuppressive potential of sCTLA-4 approached that of CTLA4-Ig, the dimeric fusion protein, which has proven clinically useful for the treatment of rheumatoid arthritis. The CTLA-4 receptor has relatively high affinity for its B7 ligands largely because it forms a functional homo-dimeric complex resulting from a disulphide bridge between cysteine residues at position 127. This residue is lost in sCTLA-4, which has led to the notion that sCTLA-4 is monomeric and therefore deprived of the potent immunosuppressive qualities of its dimeric receptor counterpart and indeed recombinant CTLA4-Ig, which is also dimeric. However, recent data suggest a novel mechanism, in which sCTLA-4 binds to the RGMB receptor enhancing both sCTLA-4 engagement with B7 ligands and its immunosuppressive action thus potentially explaining its capacity for immunosuppression (30).

An interesting corollary to this work was the observation that antibody blockade of the CTLA-4 receptor in both CD4^+^ and CD8^+^ T cells stimulated with anti-CD3 mAb, suppressed phosphorylation levels of the key T cell signalling intermediates ZAP-70 and Slp76 implying that crosslinking of CTLA-4 by antibody can to some extent at least reduce the activational capacity of individual effector T cells. This probably explains explain some of the inhibitory effects of anti-CTLA-4 antibody blockade originally observed during characterisation of CTLA-4 function (43). Antibodies that selectively target sCTLA-4 are in effect, functionally exempted from having this inhibitory capacity, because they do not bind to CTLA-4 on cells.

Previously, we identified regulatory T cells to express relatively high amounts of sCTLA-4 and set about investigating whether or not TGFβ plays a role in increasing levels of sCTLA-4. Initially our data were mixed with no clear evidence that TGFβ1 consistently contributed to induction in human T cells. Serum levels, however, of a second isoform, TGFβ2, have previously been demonstrated to correlate with disease progression from primary lesions to malignant distal metastasis (44). TGFβ2 was originally isolated from a glioblastoma cell line as Glioblastoma-derived T-cell suppressor factor (G-TSF) and since then several cancers including melanoma, have been found to secrete increased amounts of this immunosuppressive cytokine (32). Our study confirmed significantly higher serum levels of both TGFβ1 and TGFβ2 within the melanoma patient cohort, while analysis of lupus patient sera from a previous study identified high levels of TGFβ1 but little TGFβ2 compared with healthy donor sera. The correlation of sCTLA-4 with TGFβ2 serum levels exclusively in melanoma patient sera was surprising and immediately raised the notion of a potential new mechanism of immune cell evasion. This idea was supported by our data showing that a TGFβ2 based induction protocol did indeed drive both CD4^+^ and CD8^+^ T cells to produce significantly high culture supernatant levels of sCTLA-4 in both humans and mice. Collectively, these observations raise the notion that in melanoma, tumor cells can modulate sCTLA-4 production by T cells, perhaps as a mechanism of immune evasion. Our analysis of sCTLA-4 in the lupus patient cohort indicates that increased sCTLA-4 production is not, however, exclusively dependent on TGFβ2 but may be associated particularly with cancers known to secrete high levels of the TGFβ2 isoform. It is now essential that levels of both TGFβ2 and sCTLA-4 are assessed in other cancers associated with increased TGFβ2 levels, especially those that have previously been identified as “cold” tumor types (45).

We also examined whether or not selective anti-sCTLA-4 antibody blockade of sCTLA-4 influenced immune response intensity following stimulation of PBMC with increasing levels of stimulatory anti-CD3 mAb in patients suffering melanoma. Previously, we identified selective blockade of sCTLA-4 to enhance IL-17 and IFN-γ effector cytokine supernatant levels of healthy donor PBMC stimulated with recall antigens or anti-CD3 mAb (36). In a large cohort of lupus patients, however, we could not detect this cytokine enhancement (37). In this study, once again PBMC from the healthy donor cohort responded to sCTLA-4 antibody blockade as before by producing significantly increased levels of both IFN-γ and IL-17, whereas PBMC from the melanoma patient cohort was slightly less responsive to sCTLA-4 blockade with regard to IFN-γ but not IL-17. The increased effects of sCTLA-4 blockade by anti-sCTLA-4 clone 73-B1, particularly of IL-17, and to a lesser extent, IFN-γ, compared with ipilimumab arises partially because blockade with ipilimumab decreases cytokine levels slightly. This may point to a cross-linking effect in which anti-CTLA-4 antibodies rather than blocking CTLA-4 mediated inhibition, deliver an agonist inhibitory signal to the cytokine secreting effector T cell (see **Figure 2**), but this has not been confirmed. This does, however, need to be evaluated in more detail. It is likely that there are other immunosuppressive elements, e.g., IL-10 or TGFβ or PD-1 mediated inhibitory activity, influencing immune response intensity in melanoma patients. Another aspect that is being actively pursued is whether sCTLA-4 selectively inhibits the secretion of individual cytokines, or if it has general immunosuppressive effects like recombinant soluble CTLA4-Ig. Serum from patients with high levels of sCTLA-4 was immunosuppressive to healthy donor PBMC responses and was partially but not completely reversed by anti-sCTLA-4 blockade, supporting the notion that other soluble immunosuppressive factors are at play (data not shown).

Together, the data here support a role for sCTLA-4 in cancer immunoregulation, which is almost certain to influence current therapeutic approaches based on anti-CTLA-4 antibodies. Indeed, sCTLA-4 may itself form a therapeutic target.

## Data Availability Statement

The original contributions presented in the study are included in the article/**Supplementary Material**. Further inquiries can be directed to the corresponding author.

## Ethics Statement

The studies involving human participants were reviewed and approved by East of Scotland Research Ethics Committee (ref:13/NS/0126 & 10/S1401/20). The patients/participants provided their written informed consent to participate in this study. Ethical review and approval was not required for the animal study because tissues were obtained from mice already euthanised under the UK schedule 1 protocol outlined in the the Animals (Scientific Procedures) Act 1986.

## Author Contributions

RK: designed and performed research studies including acquiring and analysing data and contributed to writing the manuscript. CZ and FA-F: designed and performed research studies including acquiring and analysing data. LL: managed the clinical study including recruiting patients, maintaining patient logs and co-ordinating between the science and clinical teams. ID: contributed to study design, recruiting patient volunteers and providing clinical advice. FMe, FMu, and MN: contributed to study design, recruiting patient volunteers and providing clinical advice. LD and RA: contributed to study design and writing of the manuscript. SR: contributed to study design, recruiting patient volunteers and providing clinical advice. RB: contributed to study design and writing of the manuscript. AO: contributed to study design, co-ordinated the clinical study, ethics applications and writing of the manuscript. FW: designed and co-ordinated the study, performed research studies, analysed data and wrote the manuscript. All authors contributed to the article and approved the submitted version.

## Funding

This work was funded by the Chief Scientist’s office, Scotland grant no. ETM/280.

## Conflict of Interest

FW, RB, and LD are named inventors on patent entitled “*Antibodies specifically directed to a soluble form of CTLA-4*” (Patent No. US8697845 B2). FW is a director on the board of Aperio Pharma Ltd., which is developing anti-sCTLA-4 based antibody immunotherapy.

The remaining authors declare that the research was conducted in the absence of any commercial or financial relationships that could be construed as a potential conflict of interest.

## Publisher’s Note

All claims expressed in this article are solely those of the authors and do not necessarily represent those of their affiliated organizations, or those of the publisher, the editors and the reviewers. Any product that may be evaluated in this article, or claim that may be made by its manufacturer, is not guaranteed or endorsed by the publisher.
