# Supplementary material for: TGFβ2 Induces the Soluble Isoform of CTLA-4 – Implications for CTLA-4 Based Checkpoint Inhibitor Antibodies in Malignant Melanoma
Source: Front Immunol. 2022 Jan 5;12:763877. doi: 10.3389/fimmu.2021.763877 (PMC8767111; doi:10.3389/fimmu.2021.763877)
Supplement: Supplementary file 1 [file DataSheet_1.docx]

Supplementary Figures


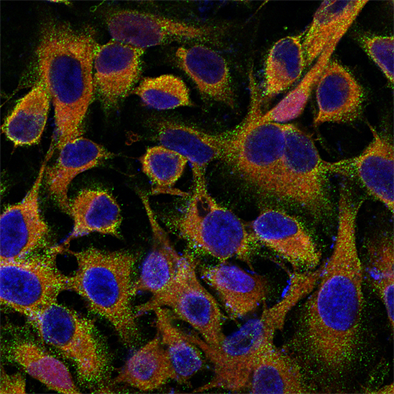

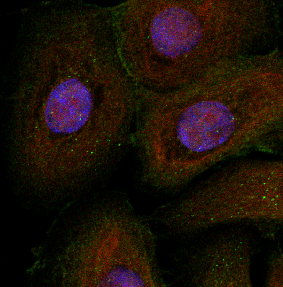


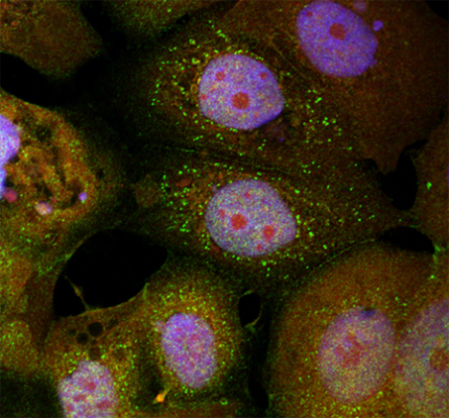

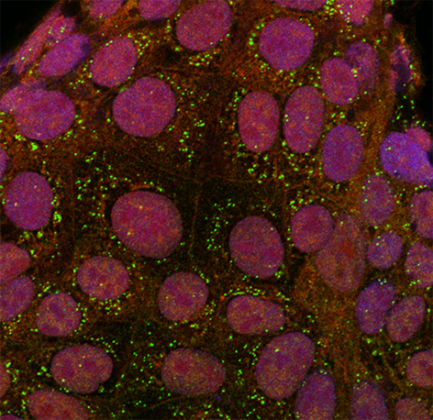


Supplementary figure 1. Cell line images that display differential CTLA-4 and sCTLA-4 staining. CTLA-4/sCTLA-4 staining in primary lung epithelial cells (top left), A549 lung adenocarcinoma cells (top right), HCT8 colon carcinoma cells (bottom left) and MC38 breast cancer adenocarcinoma cells (bottom right). Each cell line was stained with specific anti-sCTLA-4 mAb (JMW-3B3, AF555 - red) and rabbit polyclonal anti-panCTLA-4 (AF488 – green) together with DAPI (blue). Both antibodies bind sCTLA-4 yielding a yellow/orange color. See methods for staining procedure.


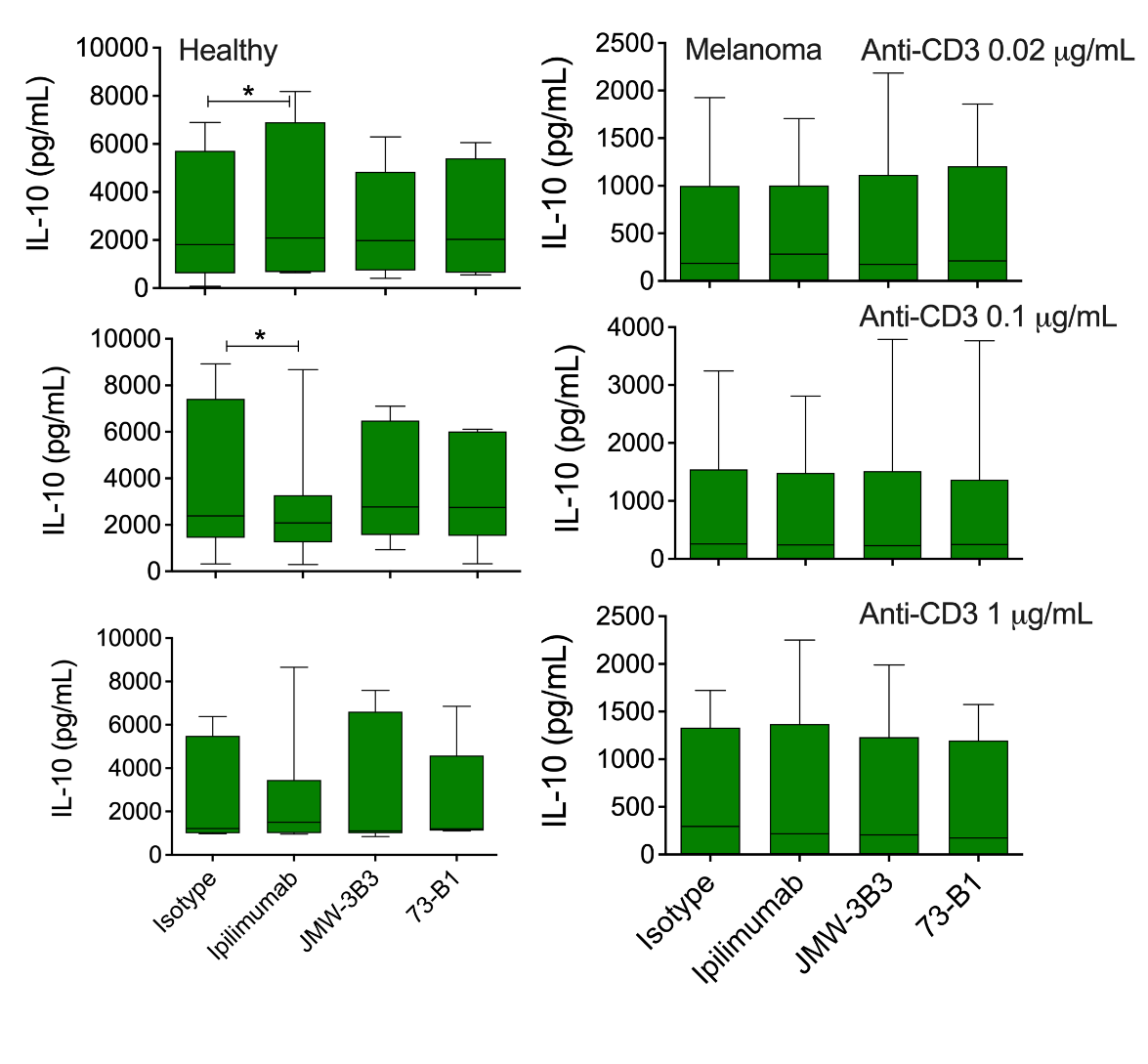


Supplementary figure 2. Comparative effect of anti-sCTLA-4 or ipilimumab anti-CTLA-4 mAb blockade on healthy donor PBMC responses to stimulation with 0, 0.1 or 1 μg/mL anti-CD3 mAb. PBMC were stimulated for five days at 37˚C 5%CO2 in the presence of plate-bound anti-CD3 and 10 μg/mL IgG1 isotype control, anti-sCTLA-4 mAbs JMW-3B3 and 73-B1, and anti-CTLA-4 mAb, ipilimumab. Cell culture supernatants were measured by ELISA for levels of IL-10. n=8; * P<0.05, P values determined a one-way test with Tukey post-hoc analysis).
